# Complete remission of an early-stage laryngeal cancer under combined pembrolizumab and chemotherapy treatment of a synchronous lung adenocarcinoma

**DOI:** 10.1186/s40463-022-00572-y

**Published:** 2022-05-16

**Authors:** Maximilian Linxweiler, Jan Philipp Kühn, Christian Neubert, Fadi Khreish, Benedikt Balensiefer, Mathias Wagner, Bernhard Schick

**Affiliations:** 1 Department of Otorhinolaryngology, Head and Neck Surgery, Saarland University Medical Center, Kirrbergerstr. 100, Building 6, 66421 Homburg/Saar, Germany; 2 Department of Nuclear Medicine, Saarland University Medical Center, Homburg/Saar, Germany; 3 Department of Hematology, Oncology, Clinical Immunology and Rheumatology, Saarland University Medical Center, Homburg/Saar, Germany; 4 Department of General and Surgical Pathology, Saarland University Medical Center, Homburg/Saar, Germany

**Keywords:** Head and neck cancer, Immunotherapy, Early-stage disease, Pembrolizumab

## Abstract

**Background:**

Anti-PD1-Checkpoint inhibition (CI) is an established treatment of recurrent and/or metastatic head and neck cancer. A potential benefit from CI in early-stage disease that is usually treated by radiation or surgery has not been investigated so far and is currently not addressed in clinical trials.

**Case presentation:**

A 58-year-old man was diagnosed with a cT2 supraglottic laryngeal cancer and a synchronous metastasized adenocarcinoma of the lung. As the patient refused any treatment of his laryngeal cancer, he received combined immune-chemotherapy according to the KEYNOTE-189 protocol. After 4 cycles of pembrolizumab/carboplatin/pemetrexed, the patient showed a complete remission of his laryngeal cancer with a clear shrinkage of the mediastinal and hilar lung cancer metastases. After 21 cycles of maintenance therapy, the lung adenocarcinoma shows a stable disease status with no signs of any residual or recurrent laryngeal cancer.

**Conclusions:**

Anti-PD1-CI may be a treatment option also for early-stage HNSCC with excellent functional outcome when established therapies are not available.

**Graphical Abstract:**

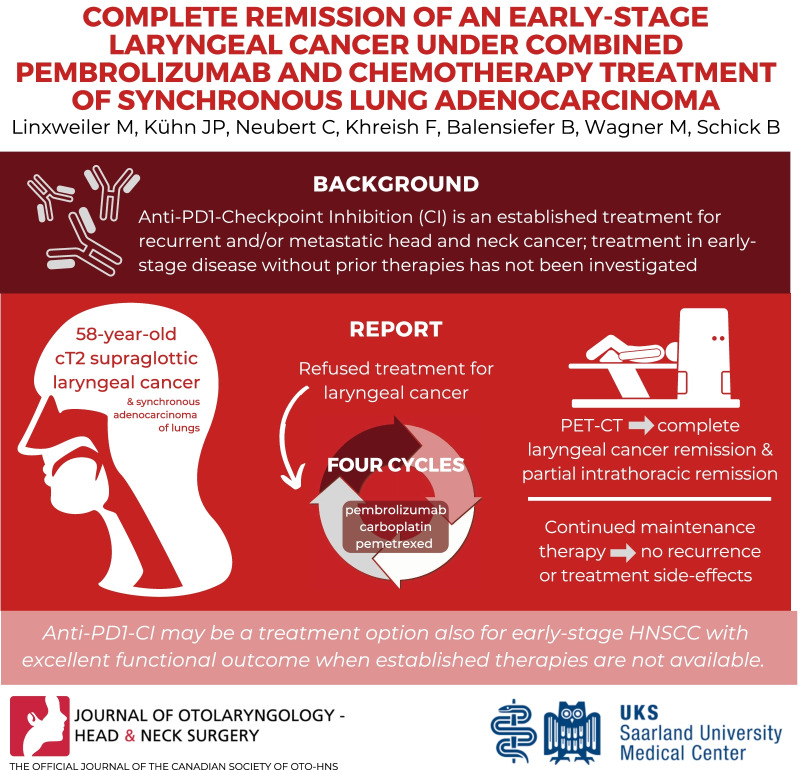

## Background

Immune checkpoint inhibition (ICI) has revolutionized our understanding of cancer biology and systemic cancer therapy and has been implemented in treatment standards of a wide spectrum of hematological and solid human malignancies [1]. Cancer immunotherapy using Anti-PD-1 antibodies is established in head and neck oncology for the first-line treatment of recurrent and/or metastatic squamous cell carcinomas (RM-HNSCC) as single therapy or in combination with platinum-based chemotherapy [2]. Additionally, anti-PD-1 antibodies are a treatment option as monotherapy in a second line scenario for RM-HNSCC patients after failure of platinum-based therapy [3, 4]. An enormous number of ongoing clinical trials is investigating further indications for ICI treatment in head and neck oncology including concepts for neoadjuvant [5, 6] and adjuvant treatment [5] as well as combinations with other immuno- and chemotherapeutics [7]. Hence, one can expect that ICI will soon challenge current treatment standards of locally-advanced non-metastasized and non-recurrent HNSCCs and possibly also early-stage HNSCCs [8, 9]. However, evident data from prospective clinical trials investigating a use of ICI for treating these cancer stages are missing so far, so that surgery, radiation and/or chemoradiation will remain standard of treatment.

In lung cancer pembrolizumab is FDA- and EMA-approved for the treatment of metastasized non-squamous non-small cell lung cancer without EGFR or ALK mutations in combination with pemetrexed and platinum-based chemotherapy [10]. As secondary malignancies are found in up to 2.5% of HNSCC patients [11] and chronic nicotine consumption represents a major risk factor for developing head and neck as well as lung cancer, a synchronous treatment of both malignancies represents a relevant challenge for clinicians of both specialities especially when multimodal treatment is indicated.

In this case report, we describe to the best of our knowledge the first case in literature of a synchronous NSCLC and early-stage HNSCC that were both successfully treated with systemic immuno-chemotherapy indicating a promising role for ICI in treatment of locally limited head and neck cancers.

## Case presentation

A 58-year-old Caucasian man presented at the Department of Otorhinolaryngology, Head and Neck Surgery with a left-sided painless cervical mass that he first recognized about 8 weeks ago as well as a persistent throat irritation. A conservative treatment with proton-pump inhibitors over 4 weeks initiated by his family doctor could not remove his symptoms. He neither reported on night-sweat, weight loss or fever nor did he have any trouble with swallowing and breathing. Pre-existing medical conditions included hypertension treated with valsartan, spondylarthrosis of the lumbar spine and a bilateral gonarthrosis. The patient reported a smoking history of 40 pack-years and occasional alcohol consumption.

A blood sample for laboratory analysis including CRP, parameters for kidney and liver dysfunction, electrolytes, and differential blood count showed no conspicuous findings. In the clinical otolaryngological examination we found an irregularly shaped tumor arising from the posterior part of the left vestibular fold with a diameter of about 10 mm and a reduced mobility of the left vocal cord. Ultrasonography of the neck showed three roundly-shaped lymph nodes with a diameter up to 1.4 mm in the left supraclavicular region. All three nodes had an irregular structure with a lack of a clear vascular hilum resulting in a cN2b neck status.

**Fig. 1:**
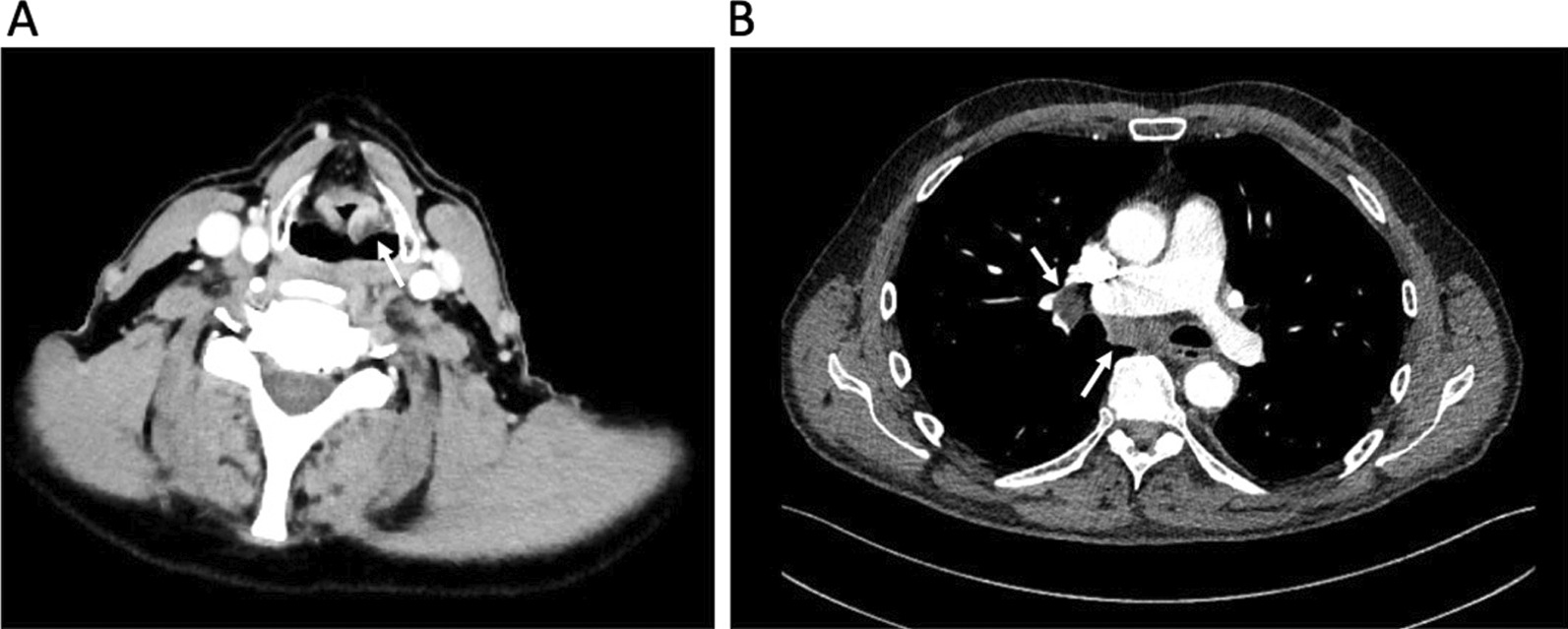
CT scans of the laryngeal cancer (**A**) and lung adenocarcinoma (**B**) before treatment start. **A** Transversal CT images of the neck showing a moderately contrast enhancing tumor of the left vestibular fold measuring 12 × 13 × 11 mm (white arrow). **B** Transversal CT scan of the thorax showing mediastinal and right hilar nodal metastases (white arrow)

**Fig. 2:**
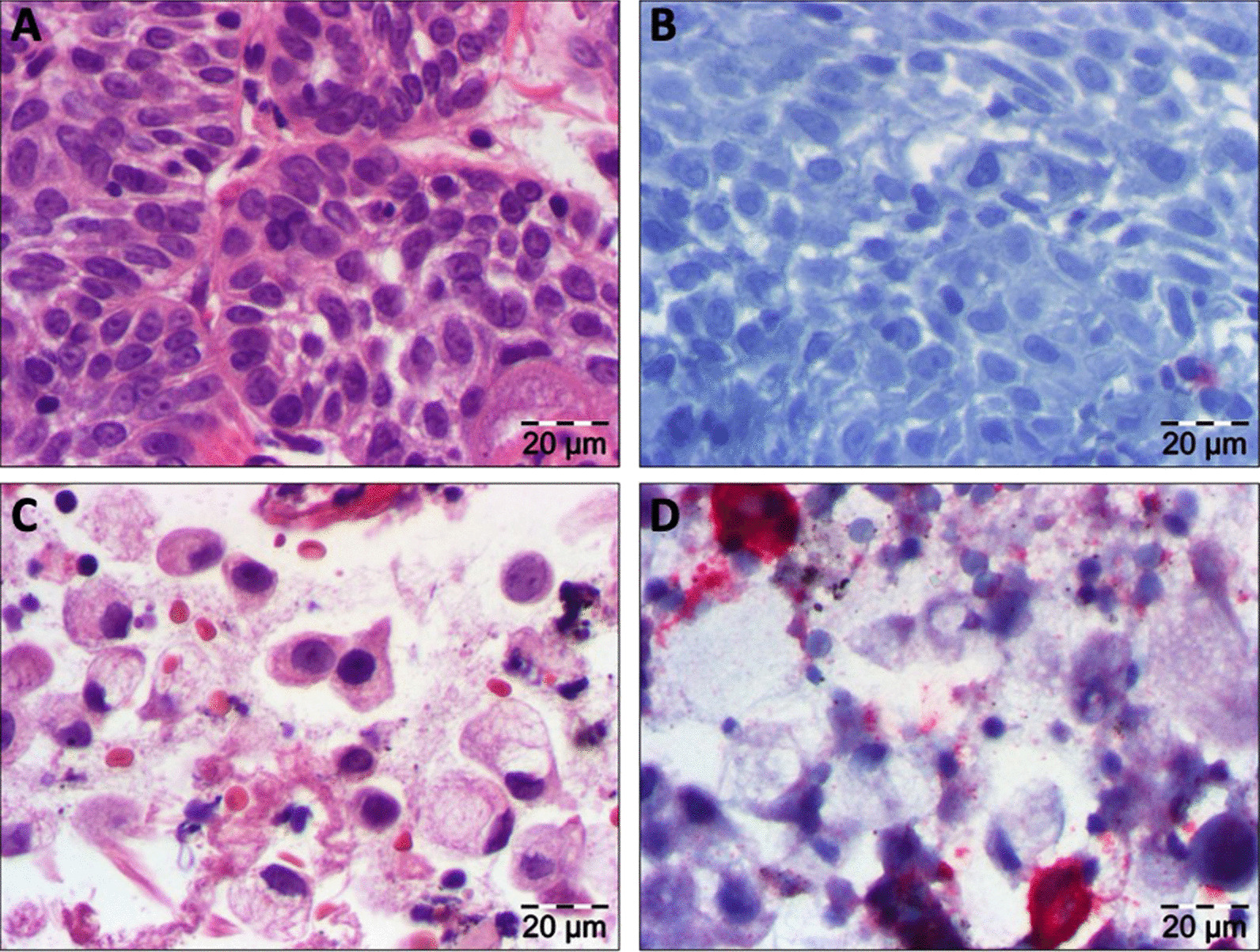
Histopathological findings of the primary tumors. **A** H&E staining of the supraglottic laryngeal cancer. **B** Anti-PD1 immunohistochemical staining (clone 22C3) of the supraglottic laryngeal cancer. **C** H&E staining of the lung adenocarcinoma. **D** Anti-PD1 immununohistochemical staining of the lung adenocarcinoma. In **B**, **D** immunohistochemical reaction is indicated by a red signal

With the clinical diagnosis of a cT2 cN2b cM0 supraglottic laryngeal cancer we indicated a CT scan of the head, neck, thorax, and abdomen that showed not only the aforementioned laryngeal mass on the left side with moderate contrast enhancement (Fig. 1A) but also markedly enlarged mediastinal and hilar lymph nodes on both sides (Fig. 1B). A whole-body 18-FDG-PET-CT-scan confirmed the suspected diagnosis of supraglottic laryngeal cancer and intrathoracic lymph node metastases with significant tracer uptake, respectively. Though the mediastinal und hilar lymph nodes were highly suspicious of being metastases no primary tumor could be detected in conventional CT-scan or PET-CT. Accordingly, a diagnostic panendoscopy was performed with a biopsy of the supraglottic tumor as well as a transbronchial biopsy of a representative left hilar lymph node. Histopathological examination of the laryngeal biopsy showed a moderately differentiated squamous cell carcinoma positive for p40 and CK5/6 and negative for p16 (Fig. 2A). Three separate rounds of anti-PD-L1 immunohistochemistry using the antibody clones “SP142”, “22C3”, and “28–8” were performed, resulting in a tumor proportion score (TPS) of 0% and a combined positivity score (CPS) of 0 for the laryngeal cancer (Fig. 2B). Transbronchial biopsy revealed the diagnosis of a poorly differentiated adenocarcinoma with some signet-ring type cells (Fig. 2C) and a specific signaling with the anti-pan-cytokeratin cocktail AE1/3 and antibodies to TTF-1. The lesion was completely non-reactive with antibodies to napsinA, p40 and CDX-2. It had a TPS of < 1% and a CPS of 10 (Fig. 2D). As in non-squamous NSCLC, druggable genetic alterations include EGFR mutations (target of EGFR TKI e.g. Osimertinib, Afatinib), ALK translocations (target of ALK inhibitors e.g. Alectinib, Brigatinib), and ROS1 translocations (target of ROS1 inhibitors e.g. Crizotinib, Entrectinib) further molecular testing was performed. Here, neither mutations in BRAF exons 11 and 15 and EGFR exons 18–21 nor ALK or ROS1 gene rearrangements and/or translocations were found.

**Fig. 3:**
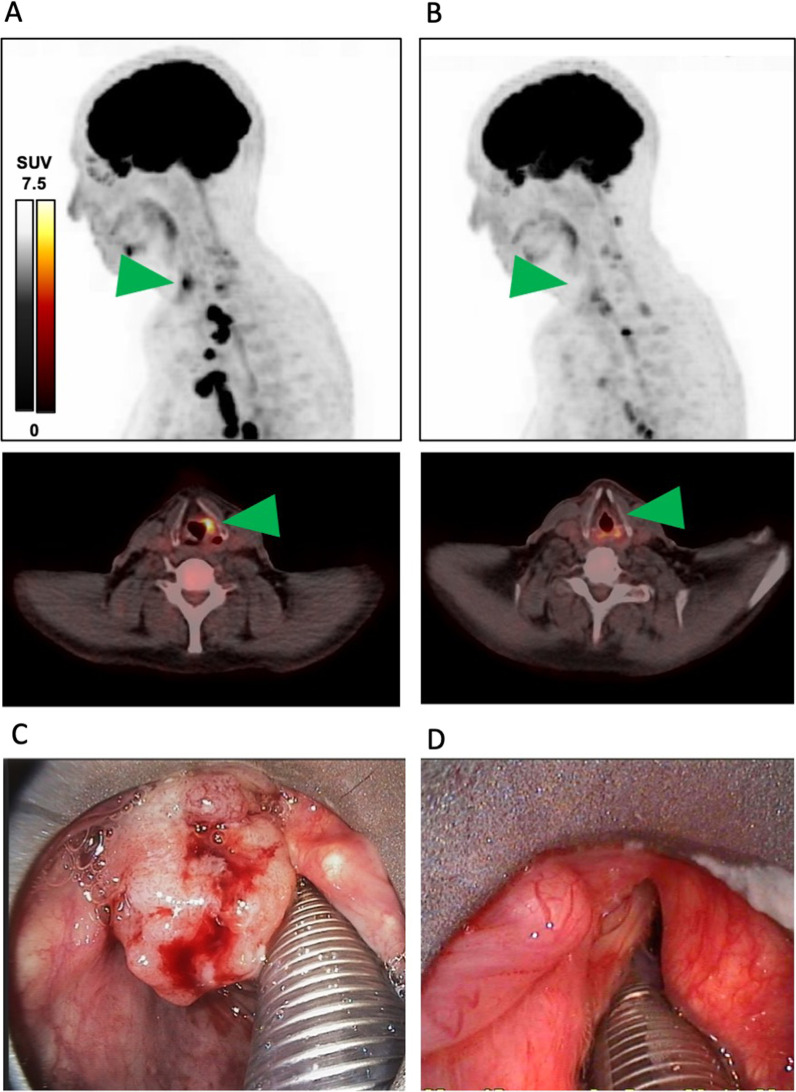
Therapy response after 4 cycles pembrolizumab/carboplatin/pemetrexed. Maximal intensity projection (MIP) of [^18^F]FDG PET/CT **A** before treatment start, **B** after 4 cycles immuno-chemotherapy. In **A**, **B** the area of the supraglottic laryngeal cancer is indicated by a green arrow. **C** Microlaryngoscopic view of the laryngeal cancer before treatment start during diagnostic panendoskopy. **D** Microlaryngoscopic view of the same area after 4 cycles immuno-chemotherapy during a control panendoscopy

Taken together, two separate malignant tumors were diagnosed: a supraglottic laryngeal squamous cell carcinoma (cT2 cN2b cM0; ICD-O: 8170/3) and a synchronous pulmonary adenocarcinoma (cTx cN3 cM0; ICD-O: 8140/3). After discussion of the case in an interdisciplinary tumor board, a transoral CO_2_ laser resection of the supraglottic laryngeal cancer was recommended followed by a systemic treatment of the lung adenocarcinoma according to the KEYNOTE-186 protocol postoperatively, which represents the first-line treatment for non-squamous NSCLC with a PD-L1 TPS < 50% [10]. However, the patient refused any kind of surgical or radiotherapeutic treatment of his laryngeal cancer after a detailed discussion of the diagnostic findings and only gave his consent for the systemic therapy regimen that was indicated for the lung adenocarcinoma. A possible benefit of the lung cancer treatment on the laryngeal cancer was not discussed due to a lack of evidence in this therapeutic setting. Accordingly, we decided to start therapy with the KEYNOTE-186 protocol under regular endoscopic controls of the laryngeal tumor. After the patient received pembrolizumab (200 mg), carboplatin (AUC 5 mg/ml/min), and pemetrexed (500 mg/m^2^) every 3 weeks for 4 cycles, another PET-CT-scan was indicated for restaging (Fig. 3).

Images showed a complete remission of the laryngeal cancer with no residual tracer uptake, which was consistent with our endoscopic findings, and a partial remission of the intrathoracic lymph nodes with size reduction in all nodes.

Treatment was continued with a maintenance therapy consisting of pembrolizumab (200 mg) and pemetrexed (500 mg/m^2^) every 3 weeks. Meanwhile, the patient received 21 cycles of maintenance therapy with an enduring complete remission of his laryngeal cancer and stable disease of mediastinal and hilar metastases. After 5 cycles of maintenance therapy, another diagnostic panendoscopy with an incisional biopsy of the former laryngeal tumor region was performed and confirmed the diagnosis of a complete remission with no detection of vital tumor cells. The patient did not show any therapy-related adverse events or side-effects over the whole treatment period.

This retrospective case study was in accordance with the ethical standards of the institutional and national research committee and with the 1964 Helsinki Declaration and its later amendments or comparable ethical standards. The patient gave his informed consent for publication of clinical data and images.

## Discussion and conclusions

Immunotherapy targeting the PD1-PD-L1 axis with monoclonal antibodies is established in the treatment of patients with recurrent and/or metastatic squamous cell carcinomas of the head and neck [2–4] and numerous ongoing clinical trials are investigating a potential use of immune checkpoint inhibitors in a neoadjuvant and adjuvant therapy setting as well as in combination with other immune therapeutics, chemotherapeutics, and radiation [5, 6, 8]. Hereby, the expression of PD-L1 represents the most evident predictive biomarker with better response rates both in the first and second-line treatment of recurrent and/or metastastic HNSCC patients (RM-HNSCC) with Pembrolizumab resulting in a clinical approval of this substance depending on aforementioned immunohistochemical scores TPS and CPS [2, 3]. Nivolumab as a second PD-1 inhibitor approved for HNSCC treatment shows a slightly different response pattern that seems to be independent of PD-L1 expression [4]. As first results of trials treating patients preoperatively with checkpoint inhibitors alone or in combination with chemotherapy reported promising data with pathologically complete responses up to 48% [12], a future paradigm shift in immunotherapy of head and neck cancers can be anticipated with indications for ICI treatment also in locally limited tumors. Some of these neoadjuvant immunotherapy trials also included resectable, early-stage HNSCC patients, e. g. the CIAO study [13], the CheckMate358 study [14], and NCT02919683 [15]. However, all of the included patients underwent tumor resection after checkpoint inhibition as a second therapeutic step. So far, there has been no reported case of a resectable, locally-limited, non-recurrent and non-metastatic (M0) head and neck cancer treated with checkpoint inhibition and chemotherapy as single treatment underlining the novelty of the reported case. In line with the results of the aforementioned neoadjuvant ICI studies in early-stage HNSCC patients this case shows that a good response can potentially be achieved in early-stage disease as well. If checkpoint inhibition with or without synchronous chemotherapy will be a therapeutic option outside a neoadjuvant setting without subsequent tumor resection has to be further investigated in clinical trials. However, as complete responses will still remain exceptional cases and the costs of ICI are much higher compared to transoral laser microsurgery or radiation that both are well tolerated in early stages of HNSCCs, one cannot recommend a routine use of ICI in this clinical setting and should only consider immunotherapy once the patient refuses any other kind of established treatment.

In view of Anti-PD-L1 immunohistochemistry results, the supraglottic laryngeal cancer as well as the lung adenocarcinoma showed comparably low PD-L1 expression on tumor and tumor-infiltrating immune cells with, however, excellent and enduring response to Anti-PD1 treatment. Hence, this case shows that in concordance with previous reports high PD-L1-TPS and CPS values increase the probability of response to pembrolizumab treatment in HNSCC patients but that there are also responders within the PD-L1 negative patients [2, 3]. In contrast, the KEYNOTE-189 study showed that in non-squamous NSCLC, response rates do not significantly differ depending on PD-L1 expression [10].

Taken together, the reported case impressively shows that ICI therapy can achieve a complete remission also in resectable locally limited head and neck cancers that would routinely undergo surgery or chemoradiation according to current treatment standards. To our knowledge this case is the first of its kind with no comparable cases reported in literature so far. As we observed an excellent functional outcome in our patient with no swallowing and speech problems, we have a first hint that ICI can potentially achieve excellent oncological outcomes with no major long-term toxicities also in early-stage disease with no further need for surgical treatment. Large-scale prospective, randomized, controlled clinical trials will have to show if ICI will occupy a place in the treatment of locally limited head and neck squamous cell carcinomas outside of a neoadjuvant approach. Based on the available clinical evidence to date, ICI as single treatment is only a therapeutic option in early-stage HNSCC cases when the patient refused any other kind of treatment including transoral laser microsurgery and radiation.

## Data Availability

Not applicable.

## Abbreviations

CPS: Combined positivity score
EMA: European Medicines Agency
FDA: Food and Drug Administration
HNSCC: Head and neck squamous cell carcinoma
ICI: Immune checkpoint inhibition
NSCLC: Non-small cell lung cancer
TPS: Tumor proportion score
